# A novel deep learning-based quantification of serial chest computed tomography in Coronavirus Disease 2019 (COVID-19)

**DOI:** 10.1038/s41598-020-80261-w

**Published:** 2021-01-11

**Authors:** Feng Pan, Lin Li, Bo Liu, Tianhe Ye, Lingli Li, Dehan Liu, Zezhen Ding, Guangfeng Chen, Bo Liang, Lian Yang, Chuansheng Zheng

**Affiliations:** 1 Department of Radiology, Union Hospital, Tongji Medical College, Huazhong University of Science and Technology, Jiefang Avenue #1277, Wuhan, 430022 China; 2 Hubei Province Key Laboratory of Molecular Imaging, Wuhan, 430022 China; 3 Shanghai Key Laboratory of Artificial Intelligence for Medical Image and Knowledge Graph, No.523 Louguanshan Road, Changning District, Shanghai, 200000 China; 4 Hangzhou YITU Healthcare Technology Co., Ltd., Shanghai, 200000 China

**Keywords:** Viral infection, Translational research

## Abstract

This study aims to explore and compare a novel deep learning-based quantification with the conventional semi-quantitative computed tomography (CT) scoring for the serial chest CT scans of COVID-19. 95 patients with confirmed COVID-19 and a total of 465 serial chest CT scans were involved, including 61 moderate patients (moderate group, 319 chest CT scans) and 34 severe patients (severe group, 146 chest CT scans). Conventional CT scoring and deep learning-based quantification were performed for all chest CT scans for two study goals: (1) Correlation between these two estimations; (2) Exploring the dynamic patterns using these two estimations between moderate and severe groups. The Spearman’s correlation coefficient between these two estimation methods was 0.920 (*p* < 0.001). predicted pulmonary involvement (CT score and percent of pulmonary lesions calculated using deep learning-based quantification) increased more rapidly and reached a higher peak on 23rd days from symptom onset in severe group, which reached a peak on 18th days in moderate group with faster absorption of the lesions. The deep learning-based quantification for COVID-19 showed a good correlation with the conventional CT scoring and demonstrated a potential benefit in the estimation of disease severities of COVID-19.

## Introduction

Coronavirus Disease 2019 (COVID-19) has become a global pandemic since the first report in December 2019 in China^1^. The global number of infections continued to grow to over 53.7 million by 15 Nov 2020, resulting in 1.3 million deaths have been reported^2^. The real-time reverse transcription-polymerase chain reaction (RT-PCR) test as the golden diagnostic modality presented a high false-negative rate of nearly one-third, which requires serial tests to avoid missed diagnosis^3,4^. Instead, the chest computed tomography (CT) demonstrated a higher sensitivity of 97% and was increasingly identified as a better screening and monitoring method in clinical practice^5,6^.

Previous studies revealed the chest CT patterns of COVID-19 with a typical change from early subpleural ground-glass opacity (GGO) to extensive consolidation, which started to be absorbed after at least 2 weeks from symptom onset^7–9^. In addition, multiple studies confirmed the correlation between higher conventional CT scores and worse prognosis of COVID-19^10–12^. However, the conventional CT scoring system is semi-quantitative and requires intensive work of the radiologists, which is challenging at the rapid increase of the infected population. On the other hand, to date, there has no ideal tool to automatically quantify viral pneumonia on CT imaging. A deep learning-based module has been set up in our center to establish a more objective and stable evaluation system for the CT estimation of the COVID-19 disease course. This study aims to explore the correlation between the conventional CT scoring system and this novel deep learning-based quantification.

## Materials and methods

This study was approved by the Ethics of Committees of Union Hospital, Tongji Medical College, Huazhong University of Science and Technology (No. 2020-0026), and followed the 1964 Helsinki Declaration and its later amendments or comparable ethical standards. Only the anonymous data was allowed to be collected and informed consent for this retrospective study was waived by Ethics of Committees of Union Hospital, Tongji Medical College, Huazhong University of Science and Technology.

### Patients and groups

931 consecutive records for patients with RT-PCR confirmed COVID-19 were reviewed retrospectively for the period from 27th January 2020 to 30th March 2020 in two newly established isolation centers (Western Campus and Zhuankou Fangcang’ Shelter Hospitals) of Union Hospital, Tongji Medical College, Huazhong University of Science and Technology. Considering the potential impact of time from symptom onset on the CT manifestations, only the recovered patients with at least three times of serial chest CT scans were involved^7,8^. Patients with a medical history of pulmonary disease or lung surgery were excluded. Patients with respiratory rate > 30 breaths/min or SpO_2_  ≤ 93% on room air were classified as severe COVID-19, otherwise as moderate COVID-19^13,14^. Patients with mechanical ventilation in the course were excluded owing to the severe moving artifacts in chest CT images. In the end, 95 patients with a total of 465 chest CT scans were involved, including 61 moderate patients (moderate group) and 34 severe patients (severe group).

### CT scan protocol

The chest CT scans were performed using a single inspiratory phase on a multi-detector CT scanner without intravenous iodine contrast injection (Philips Ingenuity Core128, Philips Medical Systems, Best, the Netherlands). The images were obtained during a single breath-hold. The fixed tube voltage was set to 120 kVp with an automatic tube current modulation. From the raw data, CT images were reconstructed with a matrix size of 512 × 512 as axial images (thickness of 1.5 mm and increment of 1.5 mm) in transverse slice orientation with iDose5 iterative reconstruction (Philips Healthcare, Best, Netherlands).

### Chest CT estimation by radiologists

The major CT demonstrations were described using internationally standard nomenclature defined by the Fleischner Society glossary and peer-reviewed literature on COVID-19, including ground-glass opacity (GGO) and consolidation^7,15–18^. A conventional semi-quantitative scoring system (CT score) was used to estimate the involved pulmonary volume of all these abnormalities^7,19^. There was a score of 0–5 corresponding to the percentage of pulmonary involvement in each lobe as: 0, 0%; 1, < 5%; 2, 6–25%; 3, 26–49%; 4, 50–75%; 5, > 75%. The scores in five lobes were summed resulting in a total CT score ranging from 0 to 25. Two experienced radiologists (BL and LY, who had 25 and 22 years of experience in thoracic radiology, respectively) performed the estimations on the institutional digital database system (Vue PACS, version 11.3.5.8902, Carestream Health, Canada) independently and a consensus was reached after their discussion if there was a disagreement. The results of chest CT evaluation using deep learning-based quantification were blinded to both radiologists.

### Chest CT evaluation using deep learning-based quantification

**Figure 1:**
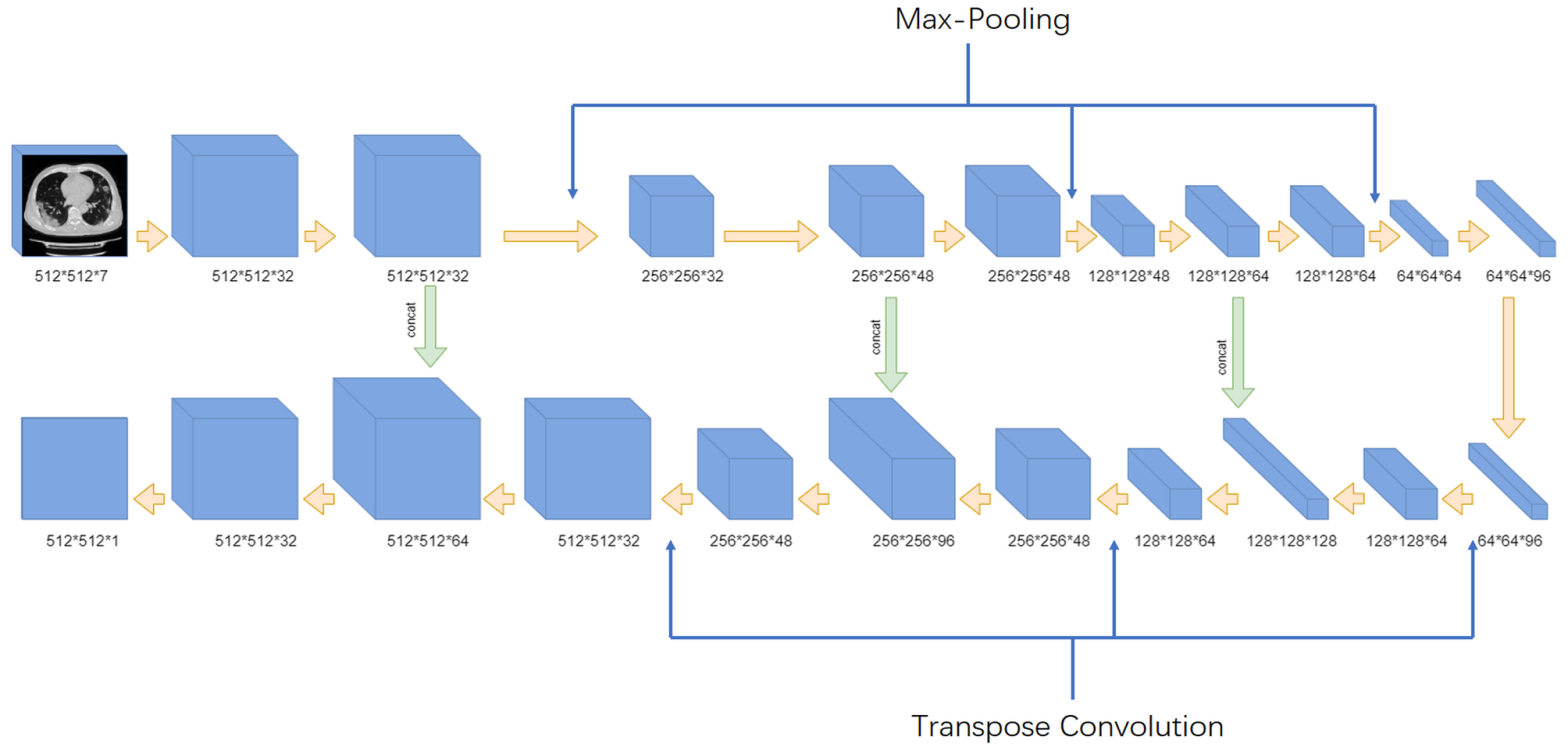
COVID-Lesion Net structure for pneumonia detection and segmentation.

The deep learning-based quantification was performed using a novel established inflammation module (COVID-Lesion Net) based on one automatic segmentation software (Yitu CT, YITU Healthcare Technology Co., Ltd., China). This module was developed as a combination of U-net and Fully convolutional networks^20–22^. In order to detect the lung lesions effectively, a contracting path and an expansive path were employed in this COVID-Lesion Net structure, which consists of three different network components: (1) Twelve convolutional segment, which included convolutional layer, batch normalization layer, and an activation layer; (2) Three max-pooling layer for down-sampling; and (3) Three transpose convolutional layer for up-sampling (Fig. 1). Information on the input CT images was passed through convolutional segments along the two paths. In addition, concatenation operations were performed between convolutional segments as bridges of contracting and expansive paths to improve the information propagation within the network. In order to train and test the COVID-Lesion Net, chest CT images without respiratory artifacts from other 942 confirmed COVID-19 patients (from 1st Jan 2020 to 1st Mar 2020) and 1340 healthy persons participating in health examinations (from 1st September 2019 to 1st November 2019) were retrospectively collected from 1st January 2020 to 1st March 2020, and randomly divided into a training set (75%) and a test set (25%) (patients not involved in this study). 100 training epochs were performed for networking training with a batch size of 8. Adam algorithm was used for the model optimizer. The ground truth region of interest (GT-ROI) for lung lesions was first drawn by a radiologist (LL with 5-year experience in thoracic radiology) and then reviewed by a senior radiologist (GC with 28-year experience in thoracic radiology), who was responsible to modify ROIs if not accepted. Dice coefficient was used to evaluate the performance of this in-house built network for both training and test set using the following equation:

$$2*\frac{overlap \; area \; of  \; PR-ROI \; and \; GT-ROI } {{\text{area}} \; \text{of} \; \text{PR}-\text{ROI}+ {\text{area} \; \text{of} \; \text{GT}-\text{ROI}}}$$

PR-ROI is the predicted ROI drawn by COVID-Lesion Net and the GT-ROI is the ROI drawn by radiologists. As a result, the Dice coefficient is 85.00% for the training set and 82.08% for the test set.

After the lesion detection, Hellinger distance and intersection over union (IOU) of lung CT distribution were calculated to reflect the differences between patients with COVID-19 and reference patients (normal CT findings in the training set)^23,24^. Quantification parameters related to lung lesions including GGO and consolidation were determined with CT value thresholds of − 750 HU and − 350 HU, respectively^25^. The bilateral lungs were also segmented by adaptive thresholding and morphological operation^26–28^. Afterwards, the volumes of bilateral lungs and pulmonary lesions including GGO, consolidation, and both were calculated. In the meanwhile, the percentages of GGO, consolidation, and both (equal to 100 × lesions volume/bilateral lung volume) were calculated as a result of “percent of GGO/consolidation/pulmonary lesions”.

### Study goals

1. Correlation between conventional CT scoring and the deep learning-based quantification;
2. Exploring the dynamic patterns using conventional CT scoring and the deep learning-based quantification between moderate and severe groups.

### Statistical analysis

Statistical analyses were performed using IBM SPSS Statistics Software (version 24; IBM, New York, USA). Quantitative data were presented as median with inter-quartile range (IQR) and frequency data were presented as the percentage of the total. The comparisons of the quantitative and counting data between moderate and severe groups were statistically evaluated using the Mann–Whitney U test and Chi-square test, respectively. The Spearman’s correlation coefficient between CT score and deep learning-based quantification assessed using deep learning-based quantification was calculated. The SPSS curve estimation module was performed to explore the optimal fitting^7^. A p-value of < 0.05 was defined as having statistical significance.

### Ethical approval

This retrospective study was approved by the Ethics of Committees of Union Hospital, Tongji Medical College, Huazhong University of Science and Technology (No. 2020-0026), and followed the 1964 Helsinki Declaration and its later amendments or comparable ethical standards.

### Patient and other consents

Informed consent/deceased patient permission form for this retrospective study was waived by the Ethics of Committees of Union Hospital, Tongji Medical College, Huazhong University of Science and Technology. Only the anonymous data was collected and analyzed to facilitate the radiological diagnosis and grading of COVID-19.

## Results

### Clinical characteristics

The details of the patients’ clinical information were summarized in Table 1. The median age of the patients was 45 years (IQR: 35–60 years) with an approximately 1:1 ratio of male to female, but the median age of severe patients was higher than moderate patients (55 years vs. 39 years) but without statistical significance. After a median of 8 days (IQR: 5–11 days) from symptom onset, patients were hospitalized. On admission, multiple abnormal biochemical and hematological parameters were observed in severe group, such as decreased lymphocyte count and elevated C-reactive protein and D-dimer. The median hospitalized period was significantly longer in severe group than in moderate group (29 days vs. 18 days). All patients underwent a median of 5 serial chest CT scans (IQR: 4–6) with a median interval of 8 days (IQR: 5–14) in the course.

**Table 1:** Basic characteristics and clinical outcomes.

	Normal reference	Total, n = 95	Moderate group, n = 61	Severe group, n = 34
**Age (year)**		45 (35–60)	39 (32–52)	55 (46–68)
**Sex**				
Male		47 (49.5)	27 (44.3)	20 (58.8)
Female		48 (50.5)	34 (55.7)	14 (41.2)
**Medical history**				
Hypertension		10 (10.5)	4 (6.6)	6 (17.6)
Diabetes		5 (5.3)	1 (1.6)	4 (11.8)
Coronary heart disease		1 (1.1)	0 (0.0)	1 (2.9)
**Initial symptoms**				
Fever		81 (85.3)	50 (82.0)	31 (91.2)
Low-grade fever (37.5–38.0 °C)		23 (24.2)	17 (27.9)	6 (17.6)
Moderate fever (38.1–39.0 °C)		35 (36.8)	23 (37.7)	12 (35.3)
High-grade fever (> 39.1 °C )		23 (24.2)	10 (16.4)	13 (38.2)
Cough		58 (61.1)	34 (55.7)	24 (70.6)
Fatigue		51 (53.7)	35 (57.4)	16 (47.1)
Expectoration		23 (24.2)	12 (19.7)	11 (32.4)
Chest distress		13 (13.7)	6 (9.8)	7 (20.6)
**Laboratory investigations on admission**				
White blood cell (G/L)	(3.50–9.50)	4.93 (3.91–6.52)	4.73 (3.78–5.84)	5.77 (4.35–7.22)
Lymphocyte (G/L)	(1.10–3.20)	1.20 (0.84–1.59)	1.36 (1.00–1.79)	0.85 (0.45–1.23)*
Hemoglobin (g/L)	(115–150)	129 (121–142)	132 (122–144)	126 (116–136)
Platelet (G/L)	(125–350)	180 (142–242)	177 (150–232)	185 (120–259)
C-reactive protein (mg/L)	(0.00–8.00)	11.90 (4.45–41.92)	7.67 (3.54–15.57)	47.63 (29.18–87.73)*
Total bilirubin (μmol/L)	(3.0–20.0)	9.6 (8.3–13.9)	9.5 (8.3–12.2)	10.4 (8.2–15.7)
Alanine aminotransferase (U/L)	(5–35)	34 (22–49)	26 (17–41)	44 (34–66)*
Aspartate aminotransferase (U/L)	(8–40)	29 (22–43)	24 (20–31)	44 (33–73)*
Lactate dehydrogenase (U/L)	(109–245)	244 (199–377)	201 (161–227)	377 (305–520)*
Serum creatinine (μmol/L)	(41.0–81.0)	71.1 (55.5–86.4)	69.5 (55.5–84.5)	71.5 (61.1–88.0)
D-dimer (mg/L)	(< 0.5)	0.40 (0.22–1.15)	0.27 (0.22–0.57)	0.77 (0.33–2.15)*
**Numbers of chest CT scans**		5 (4–6)	5 (4–6)	4 (4–5)
**Interval between two adjacent chest CT scans (day)**		8 (5–14)	8 (5–14)	8 (5–13)
**Interval between symptom onset and hospitalization (day)**		8 (5–11)	8 (5–11)	10 (5–12)
**Hospitalized period (day)**		20 (14–30)	18 (11–24)	29 (19–34)
**Period of CT follow-up from symptom onset (day)**		48 (35–60)	53 (40–66)	38 (32–50)

The comparisons of the quantitative and counting data between moderate and severe groups were statistically evaluated using the Mann–Whitney U test and Chi-square test, respectively, and the statistical difference (p < 0.05) between the two groups was noted (*).

### Correlation between conventional CT scoring and deep learning-based quantification

**Figure 2:**
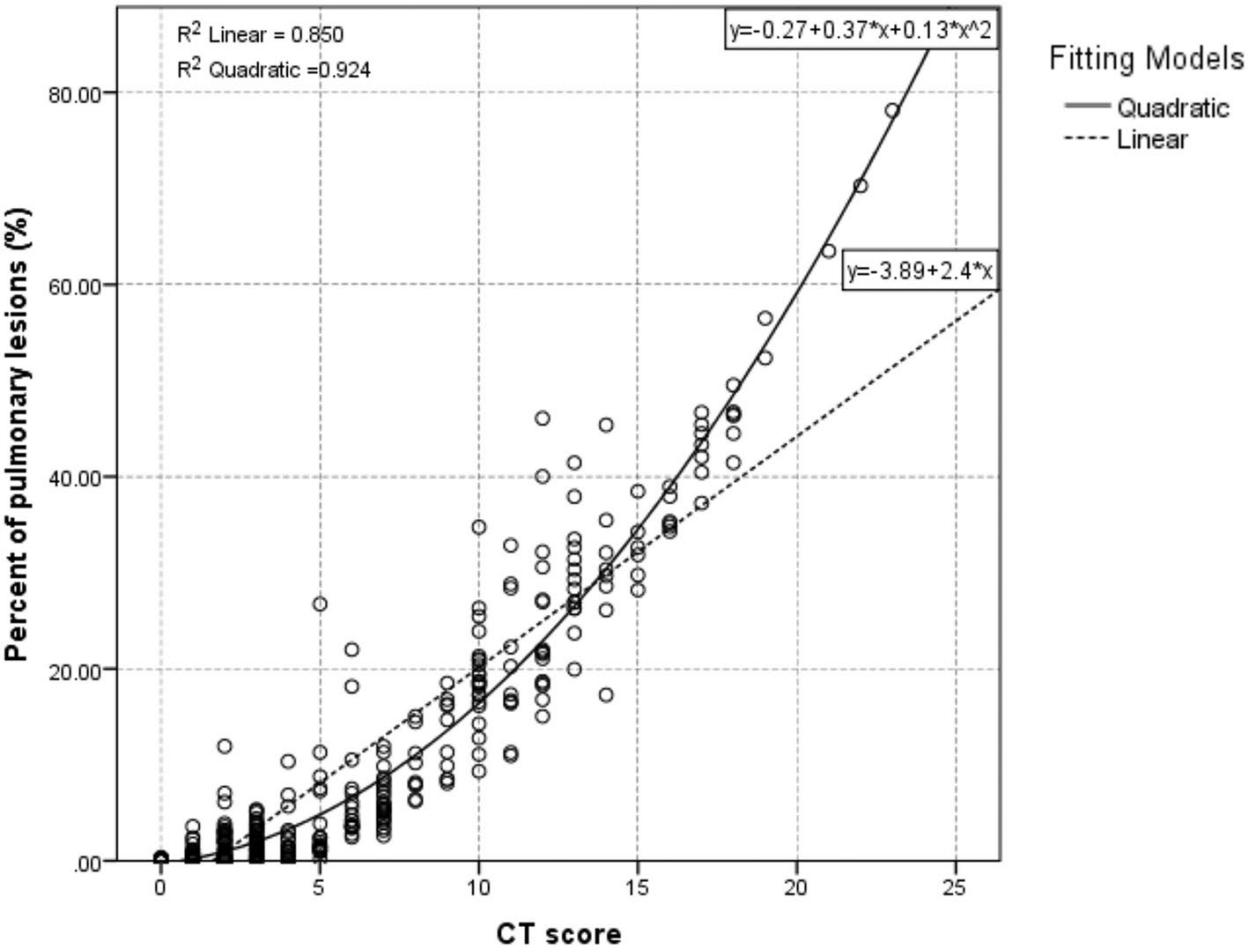
Optimal fitting between CT score and deep learning-based quantification. CT score was estimated using a conventional semi-quantitative method and percent of pulmonary lesions was calculated using deep learning-based assessment. The optimal fitting was a quadratic fitting with the equation of: $y=-0.27+0.37*x+0.13*{x}^{2}$ (*r*^2^ = 0.924, *p* < 0.001), which was better than the linear fitting ($y=-3.89+2.4*x$, *r*^2^ = 0.850, *p* < 0.001).

All 465 chest CT scans including 319 chest CT scans in moderate group and 146 chest CT scans in severe group were analyzed using conventional CT scoring and deep learning-based quantification. Based on the analysis of deep learning-based quantification, the involved patients demonstrated significant differences from the reference patients with the median Hellinger distance of 0.24 (IQR: 0.20–0.31) and the median intersection over union (IOU) of 0.66 (IQR: 0.55–0.77). In addition, GGO was identified as the major abnormal finding (median volume of 54.59 cm^3^) (Table 2). The Spearman’s correlation coefficient between CT score and percent of pulmonary lesions assessed by deep learning-based quantification was 0.920 (*p* < 0.001) (Table 3). Besides, the curve estimation presented an optimal quadratic fitting between two assessments with the *r*^2^ = 0.924, which was better than the linear fitting (*r*^2^ = 0.850) (Fig. 2).

**Table 2:** The results of deep learning-based quantification.

	Value (n = 465 scans)
Hellinger distance	0.24 (0.20–0.31)
Intersection over union (IOU)	0.66 (0.55–0.77)
Volume of bilateral lungs (cm^3^)	4036.78 (3377.81–4800.65)
Volume of pulmonary lesions (cm^3^)	69.85 (2.97–382.31)
Volume of GGO lesions (cm^3^)	54.59 (2.68–304.00)
Volume of consolidation lesions (cm^3^)	6.17 (0.25–47.42)
Percent of pulmonary lesions (%)	1.63 (0.06–10.94)
Percent of GGO lesions (%)	1.27 (0.06–8.96)
Percent of consolidation lesions (%)	0.15 (0.01–1.29)

**Table 3:** Estimations of Spearman’s correlation between CT scoring and deep learning-based quantification.

	Value (n = 465 scans)	Spearman’s Correlation coefficient	*p* value
Numbers of involved lobes—radiologists	3 (1–4)	0.959	< 0.001
Numbers of involved lobes—deep learning-based quantification	3 (1–5)		
CT score	3 (1–8)	0.920	< 0.001
Percent of pulmonary lesions (%)	1.63 (0.06–10.94)		

Controlling for the variable of "time from symptom onset (d)".

### Comparisons of conventional CT scoring and deep learning-based quantification between moderate and severe groups at different time points

The severe group presented significantly larger pulmonary lesions indicated as higher CT score and percent of pulmonary involvement calculated using deep learning-based quantification than moderate group at each time point (*p* < 0.001, each) (Table 4). Besides, the volume of bilaterally uninvolved lungs was significantly lower in severe patients compared to moderate group (Table 4). In each group at different time points, it demonstrated significant correlations between CT score and percent of pulmonary involvement assessed by deep learning-based quantification (*p* < 0.001) (Table 5). However, Spearman’s correlation coefficient was higher in severe group than in moderate group at each time point (Table 5).

**Table 4:** Comparisons of CT score and deep learning-based quantification between moderate and severe groups at different time points.

	Moderate group, n = 61 patients	Severe group, n = 34 patients	*p* value
**On admission**			
Conventional CT score	3 (1–5)	11 (5–16)	< 0.001
Deep learning-based quantification			
Volume of bilateral lungs (cm^3^)	4002.96 (3459.99–4805.01)	3378.19 (2947.54–4289.97)	0.016
Volume of pulmonary lesions (cm^3^)	51.35 (5.26–127.96)	664.50 (105.27–1238.84)	< 0.001
Volume of GGO lesions (cm^3^)	44.58 (3.91–97.92)	505.48 (87.23–1133.06)	< 0.001
Volume of consolidation lesions (cm^3^)	7.26 (0.54–28.42)	122.46 (24.44–225.26)	< 0.001
Percent of pulmonary lesions (%)	1.38 (0.12–3.18)	19.14 (3.86–40.02)	< 0.001
Percent of GGO lesions (%)	1.24 (0.11–2.83)	14.06 (2.82–35.21)	< 0.001
Percent of consolidation lesions (%)	0.18 (0.01–0.83)	3.87 (0.47–7.24)	< 0.001
**1 week after admission**			
Conventional CT score	3 (2–6)	13 (11–15)	< 0.001
Deep learning-based quantification			
Volume of bilateral lungs (cm^3^)	3947.78 (3479.82–4805.71)	3395.71 (2750.45–4043.49)	0.003
Volume of pulmonary lesions (cm^3^)	89.09 (33.37–213.00)	953.32 (608.28–1283.45)	< 0.001
Volume of GGO lesions (cm^3^)	76.55 (25.35–168.05)	751.15 (529.48–1056.68)	< 0.001
Volume of consolidation lesions (cm^3^)	13.16 (1.70–46.48)	155.00 (72.00–239.90)	< 0.001
Percent of pulmonary lesions (%)	2.31 (0.93–5.34)	28.25 (17.35–34.29)	< 0.001
Percent of GGO lesions (%)	1.85 (0.78–4.13)	22.25 (15.19–28.53)	< 0.001
Percent of consolidation lesions (%)	0.36 (0.04–1.01)	5.17 (2.27–6.87)	< 0.001
**2 weeks after admission**			
Conventional CT score	2 (1–3)	11 (7–12)	< 0.001
Deep learning-based quantification			
Volume of bilateral lungs (cm^3^)	4220.66 (3638.96–5036.93)	3390.88 (2828.49–4318.19)	< 0.001
Volume of pulmonary lesions (cm^3^)	16.98 (0.26–108.19)	635.73 (324.56–1046.70)	< 0.001
Volume of GGO lesions (cm^3^)	16.47 (0.25–91.10)	549.74 (311.01–863.06)	< 0.001
Volume of consolidation lesions (cm^3^)	1.35 (0.02–6.17)	47.67 (27.23–142.34)	< 0.001
Percent of pulmonary lesions (%)	0.46 (0.01–2.25)	18.69 (10.52–28.59)	< 0.001
Percent of GGO lesions (%)	0.41 (0.01–1.85)	17.25 (8.74–24.65)	< 0.001
Percent of consolidation lesions (%)	0.03 (0.00–0.17)	1.65 (0.72–4.51)	< 0.001

**Table 5:** Correlation at different time points in moderate and severe groups.

	Moderate group, n = 61 patients			Severe group, n = 34 patients		
	Value	Spearman’s Coefficient (*r*)	*p* value	Value	Spearman’s Coefficient (*r*)	p value
**On admission**						
CT score	3 (1–5)	0.749	< 0.001	11 (5–16)	0.954	< 0.001
Percent of pulmonary lesions (%)	1.38 (0.12–3.18)			19.14 (3.86–40.02)		
**1 week after admission**						
CT score	3 (2–6)	0.702	< 0.001	13 (11–15)	0.836	< 0.001
Percent of pulmonary lesions (%)	2.31 (0.93–5.34)			28.25 (17.35–34.29)		
**2 weeks after admission**						
CT score	2 (1–3)	0.788	< 0.001	11 (7–12)	0.830	< 0.001
Percent of pulmonary lesions (%)	0.46 (0.01–2.25)			18.69 (10.52–28.59)		

### Dynamic patterns between moderate and severe groups estimated

**Figure 3:**
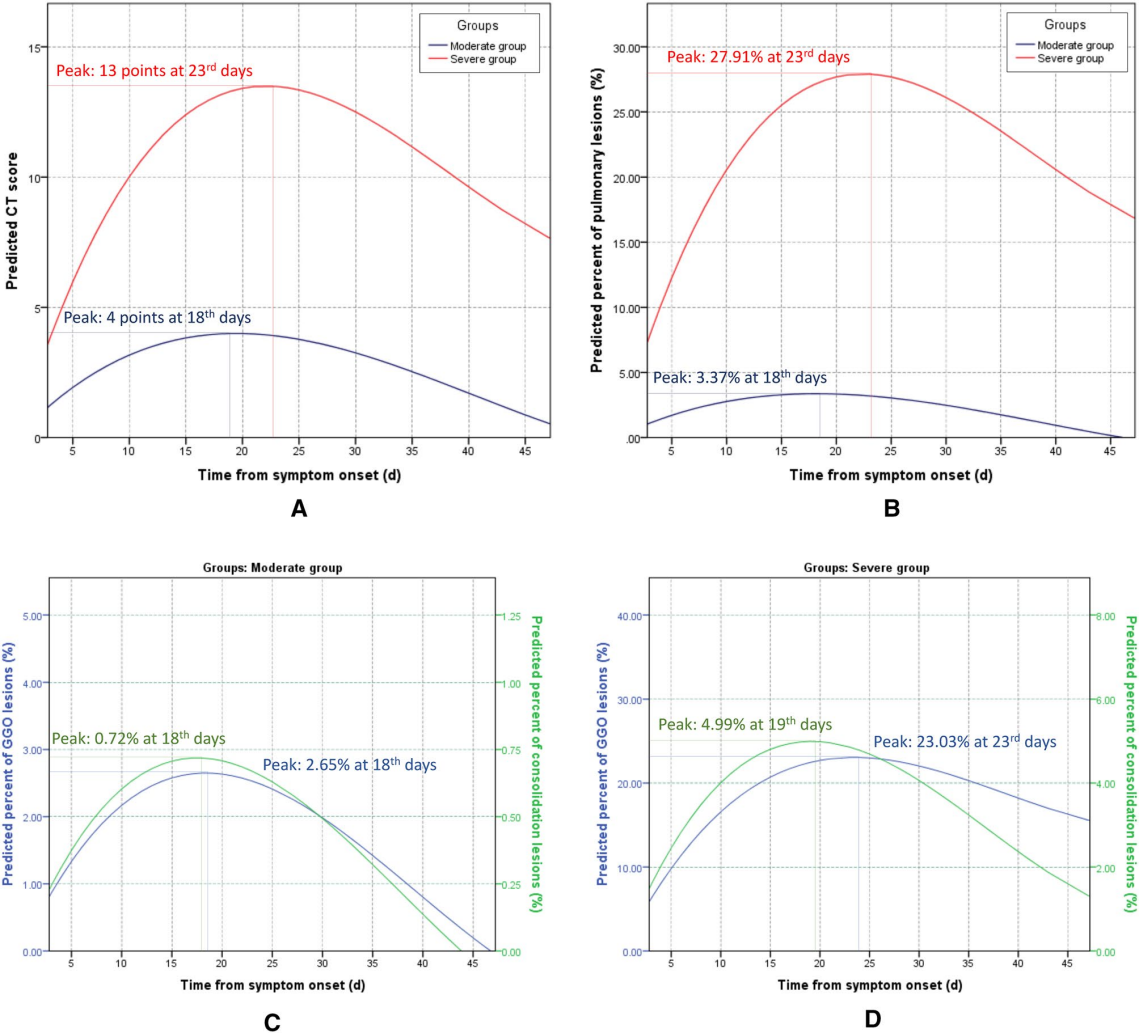
Curve estimation of dynamic patterns between moderate and severe groups. (**A**) Optimal curve fitting between conventional CT score and time from symptom onset (d) in moderate and severe groups with the equations: $y=0.460*x-0.0156*{x}^{2}+0.000128*{x}^{3}$ (*r*^2^ = 0.608, p < 0.001), and $y=1.41*x-0.0448*{x}^{2}+0.000389*{x}^{3}$ (*r*^2^ = 0.822, p < 0.001), respectively; (**B**) Optimal curve fitting between the percent of pulmonary lesions (%) calculated by deep learning-based quantification and time from symptom onset (d) in moderate and severe groups with the equations: $y=0.413*x-0.0148*{x}^{2}+0.000127*{x}^{3}$ (*r*^*2*^ = 0.319, *p* < 0.001), and $y=2.89*x-0.0912*{x}^{2}+0.000794*{x}^{3}$ (*r*^*2*^ = 0.661, *p* < 0.001), respectively. (**C**) Optimal curve fitting between the percent of pulmonary GGO lesions (%) calculated by deep learning-based quantification and time from symptom onset (d) in moderate and severe groups with the equations: $y=0.321*x-0.0114*{x}^{2}+0.0000977*{x}^{3}$ (*r*^2^ = 0.331, *p* < 0.001), and $y=3.30*x-0.0704*{x}^{2}+0.000606*{x}^{3}$ (*r*^2^ = 0.670, *p* < 0.001), respectively. (**D**) Optimal curve fitting between the percent of pulmonary consolidation lesions (%) calculated by deep learning-based quantification and time from symptom onset (d) in moderate and severe groups with the equations: $y=0.0911*x-0.00338*{x}^{2}+0.0000297*{x}^{3}$ (*r*^2^ = 0.202, *p* < 0.001), and $y=0.590*x-0.0208*{x}^{2}+0.000188*{x}^{3}$ (*r*^2^ = 0.462, *p* < 0.001), respectively.

**Figure 4:**
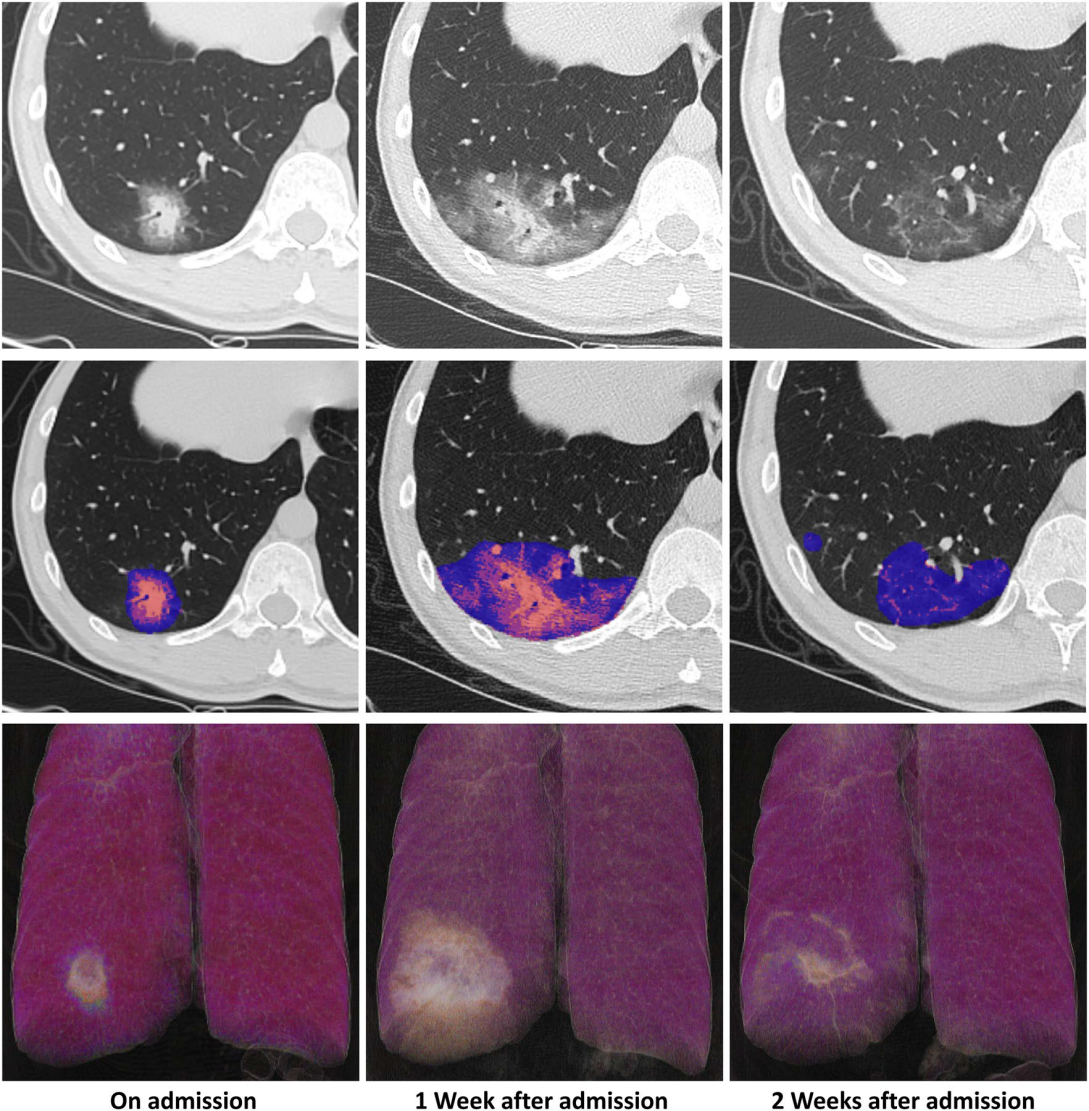
An exemplary illustration of a CT pattern in a moderate patient with COVID-19. Images from a patient presented fever for 6 days and was diagnosed with moderate COVID-19 afterwards. After admission, the serial chest CT scans were performed which demonstrated a dynamic pattern (First row) and the lesions were automatically segmented and color-coded from cold to warm color with the increase of the density using COVID-Lesion Net module (consolidation—orange; GGO—blue) (Second row). On admission (Day 6), a subpleural lesion with mixed lesions as a so-called “halo sign” [consolidation (6.56 cm^3^) and surrounding GGO (24.18 cm^3^)]. 1 week after admission (Day 13), the lesion was enlarged [consolidation (20.40 cm^3^) and surrounding GGO (133.60 cm^3^)]. 2 weeks after admission (Day 20), the lesion was partially absorbed leaving irregular residual lesions [consolidation (5.20 cm^3^) and GGO (46.74 cm^3^)]. The volume rendering images demonstrated the dynamic pattern with time more visually in which the lesions were illustrated as white color (Last row).

CT scoring and the deep learning-based quantification involving 319 chest CT scans in moderate group and 146 chest CT scans in severe group were analyzed using SPSS curve estimations, respectively. Similar patterns were observed for both groups between the predicted CT score and the percentage of pulmonary lesions calculated by deep learning-based quantification (Fig. 3A,B). The pulmonary involvement increased more rapidly and reached the peak at 23rd days from symptom onset in severe group, while, in moderate group, it reached the peak at 18th days and experienced faster absorption (Fig. 3A,B). In moderate group, the predicted percentage of GGO and consolidation lesions followed similar patterns, which reached a peak at 18th days from symptom onset (2.65% and 0.72%, respectively) and decreased afterwards (Figs. 3C and 4). But in severe group, the peaks of the predicted percentage of GGO and consolidation lesions (23.03% and 4.99%, respectively) were higher than moderate group and the consolidation started to be absorbed earlier than GGO lesions (19 days vs. 23 days from symptom onset) (Fig. 3D).

## Discussion

This study preliminarily compared a novel deep learning-based qualification to the conventional scoring system in the evaluation of COVID-19 CT manifestations. The results indicated a good correlation between these two estimations and similar findings of the CT patterns between moderate and severe COVID-19, although the correlation was relatively lower in moderate group at different time points than in the severe group. The deep learning-based qualification could calculate the percentage of the lesions separately for GGO and consolidation, which provided an added tool when compared to the conventional scoring system.

In previous studies, the CT demonstrations of COVID-19 evolved through time from symptom onset^7,8^. For example, the GGO was the major early abnormal findings but consolidation was increasingly observed with time till the start of recovery^7,8^. Therefore, irregular chest CT scans of the patients might affect the longitudinal correlation analysis between the conventional CT scoring and the deep learning-based quantification. To avoid this potential impact, only the recovered patients that had experienced serial chest CT scans with relatively regular intervals (median: 8 days) were involved. As a result, 95 patients with serial CT follow-up for more than 1 month were involved. In consistence with the previous study, severe patients presented elder age and more abnormalities of the laboratory parameters (e.g. lymphocyte count, C-reactive protein, D-dimer, etc.)^6,29–33^. Besides, moderate patients underwent more chest CT scans than severe patients resulting from the statistically longer follow-up period in moderate group compared to the severe group. However, the median interval between two adjacent chest CT scans was the same for both groups. In addition, no significant difference in the period from symptom onset to admission was found between the two groups, while the severe patients presented significantly longer hospitalized period owing to the treatment requirements. It must be pointed out that a mean of five chest CT scans was performed on each patient which brought radiation exposure issue. But under the actual pandemic pressure in that period in Wuhan, China, the shortage and high false-negative rates of the RT-PCR tests (about 2–33%) made clinical doctors chose chest CT scans as the first modality in the screening or follow-up for suspicious or confirmed COVID-19 patients which was cheaper and faster in China^4,5,13,34^. However, after the improvement of the shortage of RT-PCR tests in China, the chest CT scan was not firstly recommended at present. Thus, it will be impossible to get serial CT data like this study again.

Although some teams developed similar deep learning-based tools for the diagnosis and risk stratification of COVID-19, none was compared with the conventional radiologist-based estimation involving the whole course of this disease^35–37^. In this study, all the data of 465 serial chest CT scans were involved in the correlation analysis between conventional CT scoring and novel deep learning-based quantification. The results demonstrated a good correlation between these two estimations, not only the Spearman’s correlation analysis (*r* = 0.920, *p* < 0.001). Moreover, the optimal fitting resulted in a quadratic equation (*r*^2^ = 0.924), which was nearly linear with a relatively low slope when the CT score was less than 5 points. This may imply the risk of over-estimation of lesion areas using conventional CT scoring when the lesions were very small but distributed in multiple lobes. For instance, if there was a very small GGO in each lobe, the CT score might be 5 points, while the deep learning-based quantification could yield a lower value with higher precision. As evidence, it demonstrated a higher correlation between two methods when estimating the severe group, which presented more rapid progression and more extensive pulmonary involvement compared with moderate COVID-19 (peak percent of pulmonary lesions: 27.91% vs. 3.37%) leading to a longer disease course until the radiological resolution.

Another advantage of this deep learning-based quantification was the quantification of the lung volume and the percent of the lung involvements for different types of lesions, which was previously impossible in the context of conventional estimation by radiologists due to the extended workload, especially when mixed lesions were presented^7,8^. The novel quantification modality has enabled the dynamic pattern analysis in different groups with the precise quantification of both GGO and consolidation^25^. The quantification results of the dynamic patterns of the moderate and severe patients were similar to a cubic fitting in a previous study^7^. Furthermore, the results demonstrated that severe patients presented significantly lower lung volume than moderate patients at each time point, which might be attributed to the impairment of pulmonary function caused by COVID-19 or age factor. Therefore, the volume of bilateral lungs might correlate with the COVID-19 severities worth further exploration. On the other hand, although the predicted percent of consolidation reached the peak at a similar time (18–19 days from symptom onset) in both moderate and severe groups, the predicted peak percent of GGO and total pulmonary lesions was delayed in severe group (23 days from symptom onset, each). It was speculated that the absorption of the large area of consolidation might be accompanied by a temporal increase of GGO, reported as the “melting sugar” sign, which simultaneously demonstrated the decrease of solid components and the increase of the lesion area^1^. This phenomenon was not typical in moderate patients where the dynamic changes of GGO and consolidation seemed more synchronized. This was the major difference in the absorption stage between the two severities.

There are limitations in this study. First, although the conventional CT score that was widely used in the CT estimation of COVID-19 was chosen as the reference, to date there has been no gold standard for the lesion area quantification for viral pneumonia. Thus, whether the deep learning-based quantification is more accurate than the CT score is still uncertain. Second, all the deep-learning training and validation were from this single-center, not multi-center. Therefore, more samples from more centers are necessary for further model training to make a better model establishment.

In summary, this study evaluated a novel deep learning-based quantification for COVID-19, which showed a good correlation with the conventional CT scoring. The results indicated the potential application of deep learning-based quantification in the estimation of CT patterns and disease severities for COVID-19, and, in a broader field of view, for other types of viral pneumonia as well.

## Data Availability

The datasets generated in the current study are available from the corresponding author on request.

## Abbreviations

COVID-19: Coronavirus Disease 2019
RT-PCR: Real-time reverse transcription-polymerase chain reaction
CT: Computed tomography
GGO: Ground-glass opacity
GT-ROI: Ground truth region of interest
PR-ROI: Predicted ROI
IOU: Intersection over union
IQR: Inter-quartile range
